# The association between adverse practice experiences and residency trainee occupational burnout

**DOI:** 10.3389/fpubh.2025.1729142

**Published:** 2026-01-06

**Authors:** Qiaoying Wei, Hewei Xiao, Shenglin Liang, Ruida Zhang, Liuyan Lan, Qian Qin, Hao Yang

**Affiliations:** People's Hospital of Guangxi Zhuang Autonomous Region and Guangxi Academy of Medical Sciences, Nanning, Guangxi, China

**Keywords:** adverse practice experiences, occupational burnout, standardized resident training program, residency trainee, workplace violence

## Abstract

**Background:**

Occupational burnout among the residency trainees in the Standardized Resident Training Program is widely prevalent in China. This study investigated the current status of Adverse Practice Experiences (APEs) among residency trainees, examines their associations with occupational burnout, and ultimately proposes targeted strategies to alleviate occupational burnout and enhance training quality.

**Methods:**

The cross-sectional study used multi-stage stratified random sampling method was used to conduct an online survey of 1,328 residency trainees from 18 residency training bases in Guangxi, China. Analysis of variance was used to explore differences in occupational burnout by exposure of APEs, and multiple linear regression was conducted to examine the association of APEs and its exposure on trainees' occupational burnout.

**Results:**

The prevalence of occupational burnout among residency trainees was 70.48%. A total of 68.37% of residency trainees (908 participants) reported having encountered at least one APEs. The most common APEs were verbal abuse, which accounted for 51.51%, followed by being required to perform personal services and gender discrimination, with proportions of 49.25 and 47.14%, respectively. Residency trainees who had encountered at least one adverse practice experiences showed significantly higher occupational burnout scores (β = 0.179, *95% CI* [0.119, 0.238], *P* < 0.001). Results from the multiple linear regression analysis revealed that physical abuse (from “low exposure group” to “high exposure group”, β = 0.172–0.339, *P* < 0.001) and emotional abuse exposure (from “low exposure group” to “high exposure group”, β = 0.215–0.332, *P* < 0.001) were the most strongly associated with occupational burnout scores.

**Conclusion:**

This study finds a significant association between APEs and occupational burnout among residency trainee, but these findings do not establish causation. Residency training management departments, training bases, and trainees should collaborate to mitigate occupational burnout and foster a safer, more supportive training environment.

## 1 Introduction

The Standardized Resident Training Program (SRTP) in China has been formally established since 2014 as a kind of postgraduate education (1). This program aims to augment the professional competence and overall quality of resident physicians, aligning them with the demands of contemporary medical services (2). These professionals are required to demonstrate comprehensive mastery of medical theory and clinical practice, thereby enabling independent diagnosis and management of common diseases, prevalent conditions, and complex critical illnesses in their respective subspecialties (3). Medical students enrolled in SRTP are officially designated as residency trainees. Eligibility for residency trainee status is contingent upon fulfilling stringent admission criteria at accredited training bases, including successful completion of written examinations and structured interviews.

Trainees are required not only to fulfill rigorous clinical responsibilities but also navigate adverse events inherent to clinical environments on a daily basis. Among these challenges, Adverse Practice Experiences (APEs) are the most common negative events, including but not limited to discrimination, bullying, and harassment. Although these events have been reported internationally, research focusing on residency trainees in China has received limited attention (4–6). Empirical evidence demonstrates that APEs exert detrimental effects on trainees' career trajectory decisions (including specialty selection and residency continuation), professional identity formation, and occupational satisfaction (7–9); furthermore, these experiences may induce enduring psychological sequela.

Meanwhile, sustained exposure to clinical demands, excessive workloads, and effort-reward imbalance have been identified as key determinants of occupational burnout in residency trainees (10). Occupational burnout is defined as a state of physical and mental exhaustion, reduced enthusiasm, and disengagement resulting from prolonged exposure to high work stress and heavy workloads (11). Reports from around the world suggest that approximately one-third to one-half of physicians experience burnout (12). A cross-sectional observational study by Caesar et al. (13) found that general surgeons had the highest total burnout mean score, followed by those in emergency medicine, acute medicine, and orthopedics. In China, the prevalence of occupational burnout among residency trainees have been reported as 71.4% in Beijing, 62.2% in Shanghai, and 47.7% in Yan'an, Shaanxi Province, while the overall physicians' occupational burnout rate ranges from 66.5 to 87.8% (14–17). Additionally, one study indicated that the prevalence of occupational burnout among residents who completed the Standardized Resident Training Program was significantly lower than that of pediatric residents who had not completed the program (18).

It is worth noting that the occupational burnout experienced by residency trainees not only reflects the high-pressure clinical work environment but may also be closely related to the APEs they encounter. APEs can exacerbate occupational burnout through direct emotional distress, cumulative psychological burden, and weakened professional identity. Given that residency trainees are in a critical transitional phase from medical students to independent clinical practitioners, and considering China's unique cultural and healthcare environment, they may be more susceptible to APEs. Therefore, exploring the current situation of APEs and their impact on occupational burnout is of significant practical importance.

This study aims to reveal the current status of APEs experienced among residency trainees in Guangxi, China, and further examine how the frequency of APEs affects the level of occupational burnout among these trainees. The findings will not only provide empirical evidence to improve the quality of residency training and alleviate occupational burnout but also provide information and reference for developing targeted interventions aimed at enhancing trainees' mental health.

## 2 Methods

### 2.1 Study design and objectives

This cross-sectional study aimed to investigate the association between APEs and occupational burnout among residency trainees in Guangxi, China. The primary objectives were: (1) to describe the prevalence and types of APEs; (2) to assess the current status of occupational burnout; and (3) to quantify the association between exposure to different types and frequencies of APEs and the level of occupational burnout.

### 2.2 Data source and study sample

**Figure 1:**
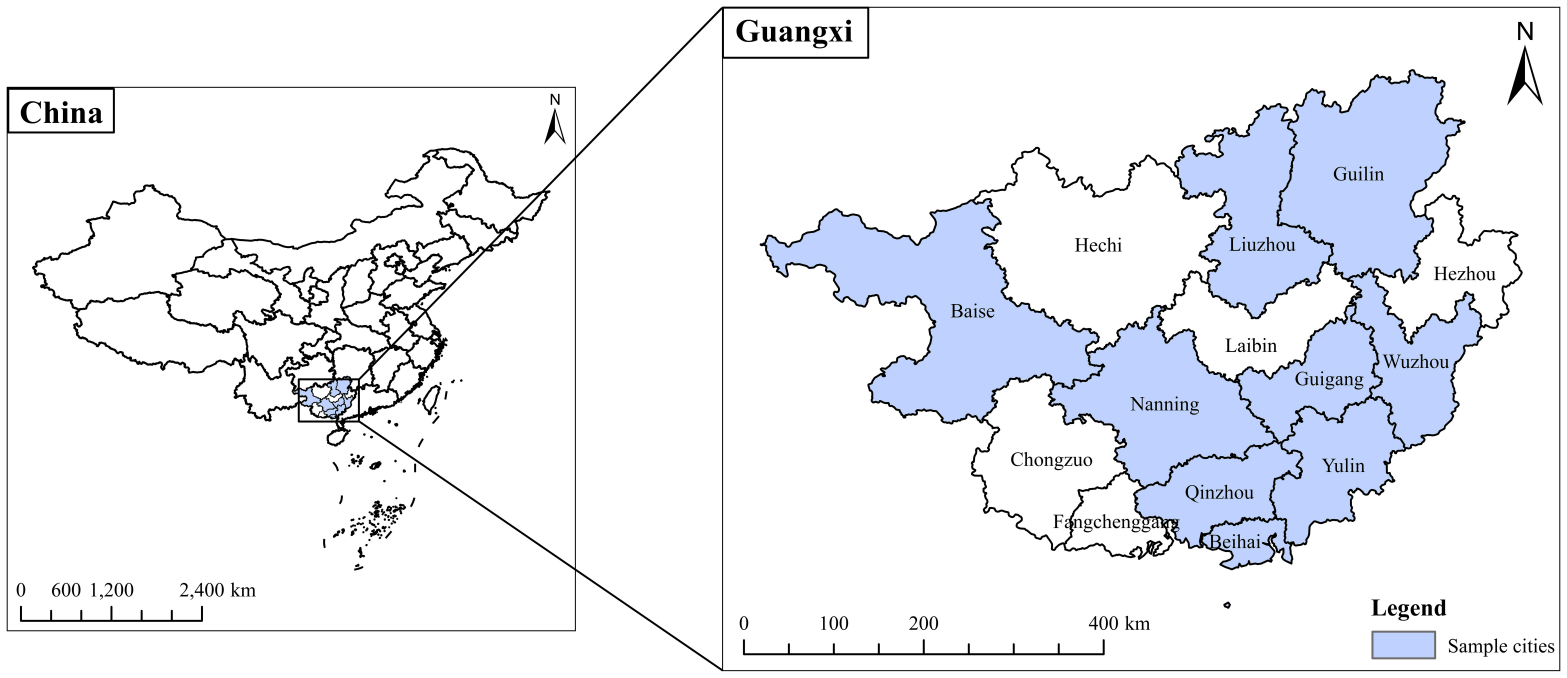
Geographical distribution of nine sample cities and prefecture in Guangxi Zhuang Autonomous Region.

A multi-stage stratified random sampling method was employed in this study. Nine prefecture-level cities of Guangxi Zhuang Autonomous Region, including Baise, Nanning, Qinzhou, Beihai, Yulin, Liuzhou, Wuzhou, Guilin, and Guigang, were selected as primary sampling units based on a comprehensive assessment of the number of residency trainees, the economic development level, and geographical location (Figure 1). In the second stage, at least one hospital with residency training qualifications was selected from each city, totaling 18 hospitals. In the third stage, adhering to the principle of voluntariness and after obtaining informed consent, residency trainees within the sampled hospitals were randomly selected by the training grade (The first year/second/third year) and specialty (e.g., Internal Medicine, Surgery, etc.) using a stratified approach, resulting in a total sample of 1,328 trainees for the survey. After obtaining informed consent, each trainee was guided to anonymously complete the questionnaire through the “Wenjuanxing” platform (a Chinese online survey platform). Data collection occurred between January 2–15, 2025. The study was approved by the People's Hospital of Guangxi Zhuang Autonomous Region and Guangxi Academy of Medical Sciences (No: KY-KJT-2023-206).

### 2.3 Adverse practice experiences measurements

Adverse Practice Experiences (APEs) refer to the non-medical negative events experienced by residency trainees during the SRTP. APEs include eight items: gender discrimination, racial/ethnic discrimination, physical abuse, verbal abuse, emotional abuse, required to perform personal services (e.g., being asked to do extra work-related matters of another person in a more senior position within a hierarchical medical system), sexual harassment, and pregnancy/childcare-related discrimination. These items were developed based on previous research and practical work experience (19–22). Responses are rated on a 7-point scale: “Never,” “Rarely,” “Occasionally,” “Often,” “Frequently,” “Very Frequently,” and “Every Day.” These responses indicate the frequency rather than the severity of these experiences. “Rarely” refers to a few times per year or less; “Occasionally” refers to once per month or less; “Often” refers to several times per month; “Frequently” refers to once per week; and “Very Frequently” refers to several times per week. We defined “Never” as the no exposure group, “Rarely” as the low exposure group, “Occasionally” as the moderate exposure group, and “Often,” “Frequently,” “Very Frequently,” and “Every Day” as the high exposure group.

To ensure the validity of APEs measurement, a pre-survey was conducted at Liuzhou Workers' Hospital in Guangxi Province (*n* = 73) prior to the formal investigation. The aim was to assess the applicability, clarity, and psychometric properties of the APEs scale among residency trainees. Exploratory Factor Analysis was performed to examine structural validity, yielding a KMO value of 0.836 and a significant Bartlett's test of sphericity (χ^2^ = 779.362, df = 28, *P* < 0.001), indicating good sampling adequacy. A single factor with an eigenvalue greater than 1 was extracted, accounting for 57.46% of the total variance, with factor loadings ranging from 0.576 to 0.840, suggesting a robust unidimensional structure. In terms of reliability, the scale showed excellent internal consistency (Cronbach's α = 0.866). These findings confirm that the APEs scale possesses satisfactory structural validity and reliability, supporting its use for assessing adverse practice experiences among residency trainees in this study.

### 2.4 Occupational burnout measurement

This study used the Maslach Burnout Inventory-General Survey (MBI-GS) scale developed by Maslach et al. (35) to assess the occupational burnout of residency trainees. The MBI-GS scale consists of 16 items and includes three dimensions: emotional exhaustion (five items), cynicism (five items), and reduced personal accomplishment (six items) (23). Chinese scholars Li Chaoping, Shi Kan, and others have adapted and revised the scale, showing that the revised version has good construct validity and internal consistency in the Chinese context (16). The MBI-GS scale is assessed using a 7-point Likert scale, with scores ranging from 0 to 6, representing the frequency of feelings (from “Never” to “Every day”). Participants self-assess based on their own experiences during the SRTP period. The Emotional Exhaustion and Cynicism dimensions are scored positively, while Reduced Personal Accomplishment is scored negatively. Based on the study by Zhang et al., occupational burnout is considered if the average score of any dimension is ≥ 3 (16). The Cronbach's α coefficient for this scale in this study was 0.863.

### 2.5 Statistical analysis

Data cleaning and analysis were conducted using STATA 18.0 statistical software. For quantitative data, descriptive statistics were presented using mean and standard deviation. For qualitative data, frequency (*n*) and proportion (%) were used for statistical description. One-way (ANOVA) and multiple linear regression analysis were used for univariate and multivariate analysis, respectively. The significance level was α = 0.05.

## 3 Results

### 3.1 Demographic characteristics

A total of 1,328 residency trainees were included in this study, with 908 in exposure group (68.37%) and 420 in no exposure group (31.63%). Of these trainees, 797 were 25 years old or younger (60.02%), 772 were female (58.13%), 907 were from rural area (68.30%). Over 70% of the trainees had a bachelor's degree or lower (Table 1).

**Table 1:** Characteristics of the study sample by exposure group (*n*, %).

**Variables**	**No exposure group**	**Exposure group**	**Total**
**Age group (years)**
≤ 25 age group	247 (18.6)	550 (41.42)	797 (60.02)
>25 age group	173 (13.03)	358 (26.96)	531 (39.98)
**Gender**
Female	199 (14.98)	573 (43.15)	772 (58.13)
Male	221 (16.64)	335 (25.23)	556 (41.87)
**Hometown**
Urban	141 (10.62)	280 (21.08)	421 (31.7)
Rural	279 (21.01)	628 (47.29)	907 (68.3)
**Marital status**
Single	395 (29.74)	866 (65.21)	1,261 (94.95)
Married	25 (1.88)	42 (3.16)	67 (5.05)
**Educational level**
Bachelor's degree and below	330 (24.85)	651 (49.02)	981 (73.87)
Pursuing a professional master's degree	55 (4.14)	174 (13.1)	229 (17.24)
Master's degree and above	35 (2.64)	83 (6.25)	118 (8.89)
**Training year**
The first year	211 (15.89)	368 (27.71)	579 (43.6)
The second year	120 (9.04)	280 (21.08)	400 (30.12)
The third year	89 (6.7)	260 (19.58)	349 (26.28)
**Fresh graduate**
Yes	104 (7.83)	175 (13.18)	279 (21.01)
No	316 (23.8)	733 (55.2)	1,049 (78.99)
**Experiencing physical fatigue due to workload**
Never	34 (2.56)	19 (1.43)	53 (3.99)
Once	27 (2.03)	31 (2.33)	58 (4.37)
Occasionally	227 (17.09)	373 (28.09)	600 (45.18)
Often	115 (8.66)	376 (28.31)	491 (36.97)
Always	17 (1.28)	109 (8.21)	126 (9.49)
**Missing or delaying personal activities due to workload**
Never	137 (10.32)	115 (8.66)	252 (18.98)
Once	32 (2.41)	49 (3.69)	81 (6.1)
Occasionally	181 (13.63)	423 (31.85)	604 (45.48)
Often	60 (4.52)	249 (18.75)	309 (23.27)
Always	10 (0.75)	72 (5.42)	82 (6.17)
**Experiencing medical disputes**
Never	115 (8.66)	74 (5.57)	189 (14.23)
Once	74 (5.57)	94 (7.08)	168 (12.65)
Occasionally	224 (16.87)	650 (48.95)	874 (65.81)
Often	6 (0.45)	85 (6.4)	91 (6.85)
Always	1 (0.08)	5 (0.38)	6 (0.45)
Total	420 (31.63)	908 (68.37)	1,328 (100)

Supplementary Table S1 presents the occupational burnout scores among trainees with different demographic characteristics. Trainees pursuing a professional master's degree exhibited the highest burnout scores. Burnout scores increased with longer duration of training. Furthermore, higher frequencies of experiencing physical fatigue due to workload, missing or delaying personal activities due to workload, and experiencing medical disputes were associated with higher burnout scores.

### 3.2 The exposure of APEs

The majority of residents in the study (*n* = 908, 68.37%) reported experiencing APEs at least once since attending SRTP. The most common APEs reported were verbal abuse (such as slander and intimidation), which accounted for 51.51%. This was followed by being required to perform personal services and gender discrimination, with proportions of 49.25 and 47.14%, respectively. 40.81% of residency trainees who experienced emotional abuse (Table 2).

**Table 2:** Characteristics of the study sample by exposure (*n* = 1,328).

**Types of APEs**	**No exposure group**	**Exposure group**			
		**Subtotal**	**Low exposure group**	**Moderate exposure group**	**High exposure group**
Gender discrimination	702 (52.86)	626 (47.14)	296 (22.29)	224 (16.87)	106 (7.98)
Racial/Ethnic discrimination	1,013 (76.28)	315 (23.72)	219 (16.49)	70 (5.27)	26 (1.96)
Physical abuse	953 (71.76)	375 (28.24)	269 (20.26)	78 (5.87)	28 (2.11)
Verbal abuse	644 (48.49)	684 (51.51)	359 (27.03)	237 (17.85)	88 (6.63)
Emotional abuse	786 (59.19)	542 (40.81)	336 (25.3)	151 (11.37)	55 (4.14)
Required to perform personal services	674 (50.75)	654 (49.25)	401 (30.2)	189 (14.23)	64 (4.82)
Sexual harassment	1,102 (82.98)	226 (17.02)	175 (13.18)	33 (2.48)	18 (1.36)
Pregnancy/Childcare-related discrimination	1,134 (85.39)	194 (14.61)	140 (10.54)	34 (2.56)	20 (1.51)

Data are presented as n (%). The percentages are calculated based on the total study sample (N = 1,328). Exposure groups were defined as follows: low exposure (“Rarely”), moderate exposure (“Occasionally”), high exposure (“Often,” “Frequently,” “Very Frequently,” and “Every Day”).

### 3.3 Survey results of occupational burnout

**Figure 2:**
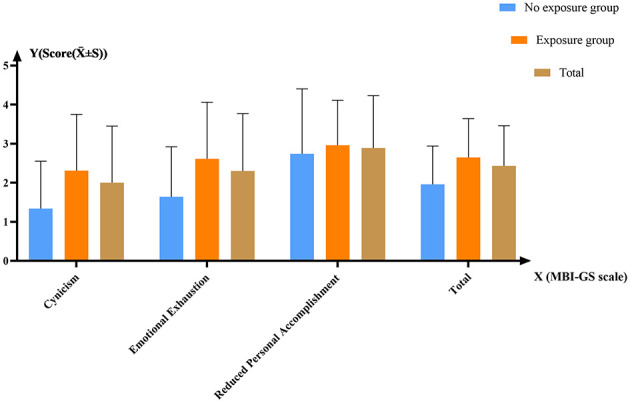
The average score for occupational burnout.

Figure 2 display the average scores of occupational burnout. The score was ≥3 points in at least one dimension for 936 trainees (70.48%), indicating that there was a high prevalence of occupational burnout. The score of emotional exhaustion was 2.30 ± 1.47, the score of cynicism was 2.00 ± 1.45, and the reverse score of reduced personal accomplishment was 2.89 ± 1.34. There were 374 residents whose average scores of two dimensions were ≥3 points and 194 residents had obvious occupational burnout in all three dimensions.

One-way ANOVA results indicated that the exposure group exhibited significantly higher occupational burnout scores than the no exposure group (*P* < 0.001). Overall, occupational burnout scores increase with higher exposure frequency. However, for sexual harassment, the high exposure group demonstrated lower occupational burnout scores than the moderate exposure group (Table 3).

**Table 3:** The average score for occupational burnout by exposure (*x* ± *s*).

**Types of discrimination**	**No exposure group**	**Exposure group**				***F***	***P***
		**Subtotal**	**Low exposure group**	**Moderate exposure group**	**High exposure group**		
Gender discrimination	2.13 ± 1.03	2.71 ± 1.42	2.57 ± 0.92	2.87 ± 0.92	3.08 ± 0.99	55.082	< 0.001
Racial/Ethnic discrimination	2.29 ± 1.04	3.22 ± 1.11	2.78 ± 0.85	3.11 ± 0.85	3.25 ± 0.78	32.090	< 0.001
Physical abuse	2.23 ± 1.02	3.22 ± 1.09	2.82 ± 0.87	3.16 ± 0.87	3.36 ± 0.99	49.377	< 0.001
Verbal abuse	2.10 ± 1.01	3.04 ± 1.11	2.51 ± 0.93	2.85 ± 0.93	3.36 ± 0.97	67.96	< 0.001
Emotional abuse	2.12 ± 1.00	3.12 ± 1.07	2.71 ± 0.87	3.07 ± 0.87	3.42 ± 0.98	79.809	< 0.001
Required to perform personal services	2.09 ± 1.00	3.01 ± 1.12	2.57 ± 0.92	3.01 ± 0.92	3.37 ± 0.96	74.317	< 0.001
Sexual harassment	2.33 ± 1.05	3.18 ± 1.12	2.84 ± 0.79	3.16 ± 0.79	3.03 ± 0.88	21.107	< 0.001
Pregnancy/Childcare-related discrimination	2.36 ± 1.05	3.18 ± 1.20	2.73 ± 0.8	3.07 ± 0.8	3.31 ± 0.82	15.579	< 0.001

### 3.4 The association between APEs and occupational burnout

Table 4 display the results of the multiple linear regression model indicated that, after controlling for confounding factors (gender, hometown, marital status, etc.), residency trainees who had encountered at least one adverse practice experiences were associated with higher occupational burnout scores (β = 0.179, *95% CI* [0.119, 0.238], *P* < 0.001). Higher frequency of experiencing physical fatigue due to workload was associated with higher occupational burnout score (from “Occasionally” to “Always,” β = 0.218–0.632, *95% CI* [0.036–0.459, 0.400–0.804] *P* < 0.05). Similarly, frequency of missing or delaying personal activities due to workload was positively associations with occupational burnout across the “Occasionally” to “Always” range (β = 0.115–0.344, *95% CI* [0.041–0.199, 0.189–0.489], *P* < 0.05). In contrast, frequency of experiencing medical disputes at the “Always” level showed no significant association with occupational burnout (β = 0.258, *P* = 0.204).

**Table 4:** Multiple linear regression analysis of factors associated with occupational burnout (*n* = 1,328).

**Characteristic**	**β**	***SE***	***t***	***P***	***95% CI***	
					**Lower**	**Upper**
**Adverse practice experiences (ref: never)**
Once	0.179	0.030	5.900	< 0.001	0.119	0.238
**Age group (ref:** ≤ **25)**
>25	−0.050	0.033	−1.500	0.134	−0.116	0.015
**Gender (ref: female)**
Male	−0.027	0.027	−1.030	0.302	−0.080	0.025
**Hometown (ref: urban)**
Rural	0.052	0.028	1.870	0.061	−0.002	0.107
**Marital status (ref: single)**
Married	0.029	0.061	0.480	0.631	−0.091	0.150
**Educational level (ref: bachelor's degree and below)**
Pursuing a professional master's degree	0.044	0.035	1.250	0.211	−0.025	0.112
Master's degree and above	−0.018	0.049	−0.360	0.719	−0.115	0.079
**Training year (ref: the first year)**
The second year	0.067	0.032	2.100	0.036	0.004	0.130
The third year	0.087	0.038	2.290	0.022	0.013	0.162
**Fresh graduate (ref: no)**
Yes	0.037	0.034	1.100	0.274	−0.030	0.105
**Experiencing physical fatigue due to workload (ref: never)**
Once	0.218	0.093	2.350	0.019	0.036	0.400
Occasionally	0.252	0.071	3.570	< 0.001	0.114	0.391
Often	0.464	0.074	6.310	< 0.001	0.320	0.609
Always	0.632	0.088	7.190	< 0.001	0.459	0.804
**Missing or delaying personal activities due to workload (ref: never)**
Once	0.084	0.063	1.330	0.183	−0.040	0.207
Occasionally	0.115	0.038	3.050	0.002	0.041	0.189
Often	0.226	0.046	4.910	< 0.001	0.135	0.316
Always	0.344	0.074	4.650	< 0.001	0.199	0.489
**Experiencing medical disputes (ref: never)**
Once	0.115	0.052	2.220	0.026	0.014	0.217
Occasionally	0.159	0.041	3.900	< 0.001	0.079	0.239
Often	0.310	0.065	4.780	< 0.001	0.182	0.437
Always	0.258	0.203	1.270	0.204	−0.141	0.657
Cons	−0.060	0.080	−0.760	0.450	−0.216	0.096

Supplementary Table S2 presents the associations between different types of APEs and occupational burnout. Overall, higher APEs exposure levels were associated with higher occupational burnout scores (*P* < 0.05). Results from the multiple linear regression analysis revealed that physical abuse (from “low exposure group” to “high exposure group,” β = 0.172–0.339, *P* < 0.001), emotional abuse (from “low exposure group” to “high exposure group,” β = 0.215–0.332, *P* < 0.001) and pregnancy/childcare-related discrimination (from “low exposure group” to “high exposure group,” β = 0.141–0.304, *P* < 0.001) were the most strongly associated with occupational burnout scores.

## 4 Discussion

To our knowledge, this is the first study to examine the association between varying levels of exposure to adverse practice experiences and occupational burnout among Chinese residency trainees. The findings' main clinical implications may help residency trainees avoid adverse clinical/medical events or errors, patient mortality, and medicolegal complaints/disputes.

The results of this study indicate that the prevalence of occupational burnout among residency trainees is 70.48%, which is higher than the prevalence reported in Shanghai (62.2%) and in Yan'an, Shaanxi (47.7%), but lower than that in Beijing (71.4%) (13–15). Compared to international findings, only 19.0% of resident physicians in France were found to experience occupational burnout (24). The possible reasons for this discrepancy may be that trainees in the study region bear a heavier workload, higher on-call frequency, or are exposed to more complex cases, leading to a higher prevalence of occupational burnout. On the other hand, cultural differences across countries may also play a role. In Western countries, a more open communication culture and work environment that emphasizes personal rights may help alleviate occupational burnout. Even though the same burnout measurement scale was used, there is still no consensus on the standard approach, which could explain the variations across studies. It is important to note that a high rate of occupational burnout not only affects the physical and mental health of trainees but also negatively impacts the development of their professional skills and the sustainability of their careers.

The study found that 68.37% of residency trainees (908 individuals) had experienced at least one APE. Specifically, the most common APE was verbal abuse (such as slander and intimidation), affecting 51.51% of trainees. This finding aligns with Crutche et al.'s (25) study in Canada; however, in Oman, the proportion increased to 88% (26). This highlights the widespread occurrence of verbal abuse during the SRTP period, with significant variation across different countries and regions. Prolonged exposure to verbal abuse was associated with higher levels of anxiety, depression, and occupational burnout, which may relate to changes in trainees' professional identity and future career choices, even suicidal thoughts (27). Additionally, verbal abuse was associated with lower self-confidence, diminish their motivation to learn, and hinder their clinical skills, ultimately compromising the quality of training.

49.25% of trainees reported being required to perform personal services, reflecting the presence of implicit power abuse during the SRTP period. Although being required to perform personal services does not involve direct verbal or physical abuse, it essentially constitutes a misuse of authority. This behavior is rooted in the long-standing hierarchical culture within the medical field, where superiors leverage their authority to demand tasks beyond the trainees' scope of learning. Trainees, being in a subordinate position, are often inclined to comply with such instructions (28). Tasks unrelated to training objectives were associated with less learning time, potentially diminish the quality of training, and are correlated with increased feelings of frustration or being undervalued among trainees. Furthermore, such practices could perpetuate a cycle of power abuse, as today's trainees may emulate these behaviors when they become superiors in the future.

A total of 47.14% of the trainees had experienced gender discrimination. Gender discrimination among medical students during their academic career was experienced significantly more often by women than men (21). In particular, males were long considered more suitable than females for surgical careers, due to traditionally accepted gender norms and stereotypes (29). Moreover, gender discrimination was associated with patterns that may contribute to persistent underrepresentation of women in certain specialties. When fewer women enter certain specialties due to discrimination, the representation of women in these fields further diminishes, reinforcing existing gender stereotypes (30).

The results of this study indicate that residency trainees who have encountered at least one APE have higher occupational burnout score. Looking at the different exposure levels of APEs, higher APEs exposure levels were associated with higher occupational burnout scores (*P* < 0.001). These findings underscore the association of APEs on the negative psychological wellbeing of residency trainees. The observed positive correlation between APEs exposure and occupational burnout aligns with prior research, which has established hostile learning environments and emotional harm as significant contributors to burnout among healthcare workers (31, 32). Chen et al. (33) find that healthcare workers with workplace violence exposure, particularly to emotional abuse, threats, and verbal sexual harassment, demonstrate higher susceptibility to burnout. This study further suggests that even limited or occasional exposure to APEs may not be benign and could have lasting psychological repercussions for residency trainees.

Due to the cross-sectional design of this study, these findings indicate associations rather than causal relationships; longitudinal studies are needed to examine potential causal mechanisms.

Burnout can lead to many challenges, including a physician's mental health issues such as suicidal tendencies and physical symptoms such as fatigue and headaches, as well as health system troubles such as poor doctor-patient relationships and clinical malpractice (15). The findings of this study have several important clinic implications for clinical education, hospital management, and resident wellbeing. Firstly, based on our findings that verbal, emotional, and physical abuse, as well as pregnancy/childcare-related discrimination, are prevalent among residency trainees, clear anti-verbal abuse policies should be formulated and enforced, establishing accessible, anonymous complaint and reporting mechanisms to enable trainees to seek help safely. Secondly, SRTP should incorporate targeted courses and workshops aimed at reducing burnout, including stress management and resilience training, mindfulness-based interventions, time management courses, and communication skills workshops for both trainees and supervisors. These initiatives are designed to foster respectful mentor-trainee relationships, improve coping strategies, and reduce conflicts and abusive behaviors. Thirdly, considering that residents reported being required to perform personal tasks unrelated to training objectives, systems must be established to clearly define the scope of responsibilities for residents, explicitly prohibiting the assignment of personal tasks unrelated to training objectives; this is crucial to combat power abuse and protect trainees' time and energy for learning. Furthermore, within the medical field, especially in traditionally male-dominated specialties such as surgery, active promotion of gender equality is essential to ensure equal opportunities in education, training, and career advancement for both women and men; increasing the visibility of female role models also helps to break down the constraints of gender stereotypes (34). Additionally, management should implement reasonable distribution of workload and on-call frequency for residents to prevent physical and mental exhaustion from overwork. Finally, it is vital to strengthen and improve accessible psychological support services for residents, ensuring they can obtain timely and effective professional help when facing stress or injustice.

## 5 Limitations

This study has several limitations. First, a retrospective approach was used to assess APEs, relying on self-reports from residency trainees, which may be subject to recall bias. Second, the generalizability of our findings may be limited. While the sampling strategy ensured good representativeness within the western coastal region of China where the study was conducted, the results may not be fully applicable to other Chinese regions with vastly different socioeconomic profiles or to distinct international medical training systems. Lastly, workload-related variables (e.g., working hours, night shifts, and training pressure) were not included as potential confounders, which may have influenced the observed associations between APEs and occupational burnout.

## 6 Conclusions

This study revealed a high prevalence of occupational burnout among residency trainees in Guangxi Province, China. A strong positive association was observed between APEs and burnout: trainees experiencing at least one APE reported significantly higher burnout levels, and increasing exposure to APEs was consistently linked to higher burnout scores. The most prevalent APEs contributing to this burden were verbal abuse, demands for personal services, and gender discrimination. It is recommended that training management departments improve relevant regulations and policies to safeguard the legal rights of residency trainees at the institutional level. Training centers should also allocate work intensity more reasonably, reduce excessive workload, and enhance attention to trainees' mental health and provide necessary interventions. At the individual level, trainees should strengthen their psychological adjustment skills, seek support and help, manage expectations appropriately, and maintain both physical and mental wellbeing.

## Data Availability

The datasets generated during and/or analyzed during the current study are available from the corresponding author on reasonable request. Requests to access these datasets should be directed to 158181313@qq.com.

## References

[1] Yang X Zheng D Wan P Luo X Zhang M Zhang L . Standard ophthalmology residency training in China: an evaluation of resident satisfaction on training program in Guangdong Province. BMC Med Educ. (2023) 23:550. doi: 10.1186/s12909-023-04527-3 37537562 PMC10401789

[2] Ye GC Liu CP Li W Zhu HC Lv L Zhu ZH . Effectiveness of multiple teaching methods in standardized training of internal medicine residents in China: a network meta-analysis. BMC Med Educ. (2025) 25:535. doi: 10.1186/s12909-025-07020-1 40234869 PMC11998199

[3] Zhu J Li W Chen L. Doctors in China: improving quality through modernisation of residency education. Lancet. (2016) 388:1922–9. doi: 10.1016/S0140-6736(16)00582-1 27339756

[4] Cormack D Gooder C Jones R Lacey C Stanley J Paine SJ . Māori medical student and physician exposure to racism, discrimination, harassment, and bullying. JAMA Network Open. (2024) 7:e2419373. doi: 10.1001/jamanetworkopen.2024.19373 38949810 PMC11217868

[5] Averbuch T Eliya Y Van Spall HGC. Systematic review of academic bullying in medical settings: dynamics and consequences. BMJ Open. (2021) 11:e043256. doi: 10.1136/bmjopen-2020-043256 34253657 PMC8311313

[6] Fnais N Soobiah C Chen MH Lillie E Perrier L Tashkhandi M . Harassment and discrimination in medical training: a systematic review and meta-analysis. Acad Med. (2014) 89:817–27. doi: 10.1097/ACM.0000000000000200 24667512

[7] Stratton TD McLaughlin MA Witte FM Fosson SE Nora LM. Does students' exposure to gender discrimination and sexual harassment in medical school affect specialty choice and residency program selection? Acad Med. (2005) 80:400–8. doi: 10.1097/00001888-200504000-00020 15793027

[8] Venkataraman S Nguyen M Chaudhry SI Desai MM Hajduk AM Mason HRC . Racial and ethnic discrimination and medical students' identity formation. JAMA Network Open. (2024) 7:e2439727. doi: 10.1001/jamanetworkopen.2024.39727 39412803 PMC11581615

[9] Pololi L Conrad P Knight S Carr P. A study of the relational aspects of the culture of academic medicine. Acad Med. (2009) 84:106–14. doi: 10.1097/ACM.0b013e3181900efc 19116486

[10] Maswadi N Khader YS Abu Slaih A. Perceived stress among resident doctors in Jordanian teaching hospitals: cross-sectional study. JMIR Public Health Surveill. (2019) 5:e14238. doi: 10.2196/14238 31579024 PMC6777282

[11] Schonfeld IS Bianchi R. From burnout to occupational depression: recent developments in research on job-related distress and occupational health. Front Public Health. (2021) 9:796401. doi: 10.3389/fpubh.2021.796401 34957039 PMC8702721

[12] Dewa CS Loong D Bonato S Thanh NX Jacobs P. How does burnout affect physician productivity? A systematic literature review. BMC Health Serv Res. (2014) 14:325. doi: 10.1186/1472-6963-14-325 25066375 PMC4119057

[13] Caesar B Barakat A Bernard C Butler D. Evaluation of physician burnout at a major trauma centre using the Copenhagen burnout inventory: cross-sectional observational study. Ir J Med Sci. (2020) 189:1451–6. doi: 10.1007/s11845-020-02223-5 32285375

[14] Lo D Wu F Chan M Chu R Li D. A systematic review of burnout among doctors in China: a cultural perspective. Asia Pac Fam Med. (2018) 17:3. doi: 10.1186/s12930-018-0040-3 29449785 PMC5806482

[15] Huang L Caspari JH Sun X Thai J Li Y Chen FZ . Risk and protective factors for burnout among physicians from standardized residency training programs in Shanghai: a cross-sectional study. BMC Health Serv Res. (2020) 20:965. doi: 10.1186/s12913-020-05816-z 33087121 PMC7576715

[16] Zhang Y Huang X Li H Zeng X Shen T. Survey results of job status of residents in a standardized residency training program. BMC Med Educ. (2019) 19:281. doi: 10.1186/s12909-019-1718-4 31345190 PMC6659202

[17] An J Chang Y Zhang X Zhang M Lei X Yang M . Status quo and influencing factors of job burnout among residents in standardized training. Front Public Health. (2024) 12:1470739. doi: 10.3389/fpubh.2024.1470739 39737464 PMC11683051

[18] Du Y Qiao L Dong L Wan C Yang X Liu H. The relationship between self-efficacy, resilience, and job burnout in pediatric residents: a cross-sectional study in Western China. BMC Med Educ. (2024) 24:787. doi: 10.1186/s12909-024-05700-y 39044219 PMC11264473

[19] Bruce AN Battista A Plankey MW Johnson LB Marshall MB. Perceptions of gender-based discrimination during surgical training and practice. Med Educ Online. (2015) 20:25923. doi: 10.3402/meo.v20.25923 25652117 PMC4317470

[20] Romanski PA Bartz D Pelletier A Johnson NR. The “invisible student”: neglect as a form of medical student mistreatment, a call to action. J Surg Educ. (2020) 77:1327–30. doi: 10.1016/j.jsurg.2020.05.013 32507361

[21] Stock J Kaifie A. The effects of gender discrimination on medical students' choice of specialty for their (junior) residency – a survey among medical students in Germany. BMC Med Educ. (2024) 24:601. doi: 10.1186/s12909-024-05579-9 38816875 PMC11140860

[22] Kloos J Simon E Sammarco A El-Nashar S Bazella C. Neglect as an undefined and overlooked aspect of medical student mistreatment: a systematic review of the literature. Med Teach. (2023) 45:1395–403. doi: 10.1080/0142159X.2023.2218982 37300429

[23] Xiong Q Luo F Chen Y Duan Y Huang J Liu H . Factors influencing fatigue, mental workload and burnout among Chinese health care workers during public emergencies: an online cross-sectional study. BMC Nurs. (2024) 23:428. doi: 10.1186/s12912-024-02070-0 38918772 PMC11197284

[24] Cohen Aubart F Lhote R Steichen O Roeser A Otriv N Levesque H . Workload, well-being and career satisfaction among French internal medicine physicians and residents in 2018. Postgrad Med J. (2020) 96:21–7. doi: 10.1136/postgradmedj-2019-136657 31467142

[25] Crutcher RA Szafran O Woloschuk W Chatur F Hansen C. Family medicine graduates' perceptions of intimidation, harassment, and discrimination during residency training. BMC Med Educ. (2011) 11:88. doi: 10.1186/1472-6920-11-88 22018090 PMC3258190

[26] Al-Shafaee M Al-Kaabi Y Al-Farsi Y White G Al-Maniri A Al-Sinawi H . Pilot study on the prevalence of abuse and mistreatment during clinical internship: a cross-sectional study among first year residents in Oman. BMJ Open. (2013) 3:e002076. doi: 10.1136/bmjopen-2012-002076 23396558 PMC3585976

[27] Hu Y-Y Ellis Ryan J Hewitt DB Yang AD Cheung EO Moskowitz JT . Discrimination, abuse, harassment, and burnout in surgical residency training. N Engl J Med. (2019) 381:1741–52. doi: 10.1056/NEJMsa1903759 31657887 PMC6907686

[28] Ma X Shen Z Xiao R Wu H. Perceived mistreatment and professional identity of medical students in China. JAMA Network Open. (2024) 7:e2444245. doi: 10.1001/jamanetworkopen.2024.44245 39514223 PMC11549658

[29] Saadé S Delafontaine A Cattan J Celanie D Saiydoun G. Attractiveness and gender dynamics in surgical specialties: a comparative analysis of French medical graduates (2017-2022). BMC Med Educ. (2024) 24:197. doi: 10.1186/s12909-024-05174-y 38413964 PMC10900538

[30] Wainwright D Harris M Wainwright E. Trainee doctors' perceptions of the surgeon stereotype and its impact on professional identification: a qualitative study. BMC Med Educ. (2022) 22:702. doi: 10.1186/s12909-022-03765-1 36195864 PMC9533602

[31] Karim Alenezi N Hamad Alyami A Omar Alrehaili B Adnan Arruhaily A Kareem Alenazi N Abdo Radman Al-Dubai S. Prevalence and associated factors of burnout among Saudi resident doctors: a multicenter cross-sectional study. Alpha Psychiatry. (2022) 23:173–83. doi: 10.5152/alphapsychiatry.2022.21361 36425745 PMC9590646

[32] Boyle AB Shay D Martyn T Savage E MacLean SBM Every-Palmer S. Burnout in New Zealand resident doctors: a cross-sectional study of prevalence and risk factors. BMJ Open. (2025) 15:e089034. doi: 10.1136/bmjopen-2024-089034 39819936 PMC11751903

[33] Chen Z Peng K Liu X Yang J Long L Liu Y . Association between high burn-out and workplace violence among healthcare workers in China: a WeChat-based survey. BMJ Open. (2022) 12:e064729. doi: 10.1136/bmjopen-2022-064729 36379659 PMC9668040

[34] Zhou L Liu B Fu W Wu W Wang Y Ju P . Surgical career choices of medical students in China: does gender bias play a role? BMC Med Educ. (2022) 22:378. doi: 10.1186/s12909-022-03453-0 35581632 PMC9112434

[35] Soares JP Lopes RH Mendonça PBS Silva CRDV Rodrigues CCFM Castro JL. Use of the maslach burnout inventory among public health care professionals: scoping review. JMIR Ment Health. (2023) 10:e44195. doi: 10.2196/44195 37477960 PMC10403803

