# Supplementary material for: The association between adverse practice experiences and residency trainee occupational burnout
Source: Front Public Health. 2026 Jan 6;13:1729142. doi: 10.3389/fpubh.2025.1729142 (PMC12816244; doi:10.3389/fpubh.2025.1729142)
Supplement: Supplementary file 1 [file Table_1.docx]

Table S1 Occupational burnout score differences stratified by demographic characteristics.

| Variables | $\bar{\text{x}}$ | *s* | t/F | *P* |
| --- | --- | --- | --- | --- |
| Age group(years) |  |  | 0.255 | 0.799 |
| ≤25 | 2.44 | 0.04 |  |  |
| ＞25 | 2.42 | 1.04 |  |  |
| Gender |  |  | 1.399 | 0.162 |
| Female | 2.46 | 0.04 |  |  |
| Male | 2.38 | 0.05 |  |  |
| Hometown |  |  | -1.837 | 0.066 |
| Urban | 2.35 | 0.05 |  |  |
| Rural | 2.47 | 0.03 |  |  |
| Marital status |  |  | 0.936 | 0.349 |
| Single | 2.44 | 0.03 |  |  |
| Married | 2.31 | 0.13 |  |  |
| Educational Level |  |  | 10.279^a^ | <0.001 |
| Bachelor’s degree and below | 2.37 | 1.03 |  |  |
| Pursuing a professional master’s degree | 2.71 | 1.07 |  |  |
| Master’s degree and above | 2.37 | 0.95 |  |  |
| Training year |  |  | 14.880^a^ | <0.001 |
| The first year | 2.27 | 1.00 |  |  |
| The second year | 2.49 | 1.02 |  |  |
| The third year | 2.63 | 1.07 |  |  |
| Fresh Graduate |  |  | 7.766 | 0.005 |
| Yes | 2.47 | 1.01 |  |  |
| No | 2.28 | 1.11 |  |  |
| Experiencing physical fatigue due to workload |  |  | 124.425^a^ | <0.001 |
| Never | 1.56 | 1.02 |  |  |
| Once | 1.91 | 0.97 |  |  |
| Occasionally | 2.04 | 0.85 |  |  |
| Often | 2.74 | 0.86 |  |  |
| Always | 3.68 | 1.02 |  |  |
| Missing or delaying personal activities due to workload |  |  | 101.550^a^ | <0.001 |
| Never | 1.90 | 0.98 |  |  |
| Once | 2.09 | 0.89 |  |  |
| Occasionally | 2.25 | 0.86 |  |  |
| Often | 2.92 | 0.92 |  |  |
| Always | 3.83 | 0.95 |  |  |
| Experiencing medical disputes |  |  | 48.031^a^ | <0.001 |
| Never | 1.88 | 1.00 |  |  |
| Once | 2.15 | 0.98 |  |  |
| Occasionally | 2.49 | 0.96 |  |  |
| Often | 3.40 | 0.99 |  |  |
| Always | 4.21 | 0.56 |  |  |

Note：a denotes the F value.

Table S2 Different types of APEs associated with occupational burnout.

| Characteristic | Model 1 Gender discrimination | Model 2 Racial/Ethnic discrimination | Model 3 Physical abuse | Model 4 Verbal abuse | Model 5 Emotional abuse | Model 6 Required to perform personal services | Model 7 Sexual harassment | Model 8 Pregnancy/Childcare-related discrimination |
| --- | --- | --- | --- | --- | --- | --- | --- | --- |
| Adverse Practice Experiences (ref:No exposure group). | | | | | | | | |
| Low exposure group | 0.159*** | 0.175*** | 0.172*** | 0.140*** | 0.215*** | 0.157*** | 0.137*** | 0.141*** |
|  | (-0.034) | (-0.036) | (-0.033) | (-0.032) | (-0.031) | (-0.031) | (-0.039) | (-0.043) |
| Moderate exposure group | 0.226*** | 0.204*** | 0.239*** | 0.199*** | 0.252*** | 0.212*** | 0.219*** | 0.161* |
|  | (-0.038) | (-0.059) | (-0.057) | (-0.037) | (-0.043) | (-0.041) | (-0.084) | (-0.083) |
| High exposure group | 0.202*** | 0.286*** | 0.339*** | 0.259*** | 0.332*** | 0.240*** | 0.289** | 0.304*** |
|  | (-0.052) | (-0.094) | (-0.091) | (-0.057) | (-0.067) | (-0.064) | (-0.113) | (-0.108) |
| Age group(ref: ≤ 25 ) | | | | | | | | |
| >25 | -0.047 | -0.051 | -0.053 | -0.054 | -0.042 | -0.047 | -0.055 | -0.053 |
|  | (-0.033) | (-0.033) | (-0.033) | (-0.033) | (-0.033) | (-0.033) | (-0.034) | (-0.034) |
| Gender(ref:Female) | | | | | | | | |
| Male | -0.003 | -0.048* | -0.049* | -0.039 | -0.041 | -0.050* | -0.043 | -0.053** |
|  | (-0.027) | (-0.026) | (-0.026) | (-0.026) | (-0.026) | (-0.026) | (-0.027) | (-0.027) |
| Hometown(ref:Urban) | | | | | | | | |
| Rural | 0.056** | 0.053* | 0.053* | 0.045 | 0.041 | 0.04 | 0.056** | 0.050* |
|  | (-0.028) | (-0.028) | (-0.028) | (-0.028) | (-0.027) | (-0.028) | (-0.028) | (-0.028) |
| Marital status(ref:Single) | | | | | | | | |
| Married | 0.025 | 0.02 | 0.029 | 0.024 | 0.013 | 0.036 | 0.025 | 0.005 |
|  | (-0.061) | (-0.061) | (-0.061) | (-0.061) | (-0.06) | (-0.061) | (-0.062) | (-0.062) |
| Educational Level(ref:Bachelor's degree and below) | | | | | | | | |
| Pursuing a professional master's degree | 0.045 | 0.04 | 0.043 | 0.041 | 0.03 | 0.038 | 0.047 | 0.05 |
|  | (-0.035) | (-0.035) | (-0.035) | (-0.035) | (-0.034) | (-0.035) | (-0.035) | (-0.035) |
| Master's degree and above | -0.03 | -0.029 | -0.028 | -0.032 | -0.025 | -0.016 | -0.017 | -0.02 |
|  | (-0.049) | (-0.049) | (-0.049) | (-0.049) | (-0.049) | (-0.049) | (-0.05) | (-0.05) |
| Grade(ref:The first grade) | | | | | | | | |
| The second grade | 0.070** | 0.075** | 0.065** | 0.058* | 0.064** | 0.061* | 0.065** | 0.067** |
|  | (-0.032) | (-0.032) | (-0.032) | (-0.032) | (-0.032) | (-0.032) | (-0.032) | (-0.032) |
| The third grade | 0.083** | 0.099*** | 0.093** | 0.081** | 0.075** | 0.080** | 0.090** | 0.093** |
|  | (-0.038) | (-0.038) | (-0.038) | (-0.038) | (-0.038) | (-0.038) | (-0.038) | (-0.038) |
| Fresh Graduate(ref:No) | | | | | | | | |
| Yes | 0.039 | 0.035 | 0.036 | 0.039 | 0.035 | 0.034 | 0.036 | 0.041 |
|  | (-0.034) | (-0.034) | (-0.034) | (-0.034) | (-0.034) | (-0.034) | (-0.034) | (-0.034) |
| Experiencing physical fatigue due to workload(ref:Never) | | | | | | | | |
| Once | 0.218** | 0.210** | 0.220** | 0.225** | 0.227** | 0.208** | 0.202** | 0.206** |
|  | (-0.092) | (-0.093) | (-0.092) | (-0.092) | (-0.091) | (-0.093) | (-0.094) | (-0.094) |
| Occasionally | 0.253*** | 0.256*** | 0.256*** | 0.245*** | 0.253*** | 0.257*** | 0.262*** | 0.261*** |
|  | (-0.071) | (-0.071) | (-0.07) | (-0.071) | (-0.07) | (-0.071) | (-0.071) | (-0.071) |
| Often | 0.461*** | 0.469*** | 0.473*** | 0.454*** | 0.468*** | 0.463*** | 0.485*** | 0.481*** |
|  | (-0.073) | (-0.073) | (-0.073) | (-0.073) | (-0.072) | (-0.073) | (-0.074) | (-0.074) |
| Always | 0.622*** | 0.652*** | 0.633*** | 0.625*** | 0.629*** | 0.638*** | 0.654*** | 0.653*** |
|  | (-0.088) | (-0.088) | (-0.087) | (-0.088) | (-0.086) | (-0.088) | (-0.088) | (-0.088) |
| Missing or delaying personal activities due to workload(ref:Never) | | | | | | | | |
| Once | 0.088 | 0.088 | 0.076 | 0.081 | 0.071 | 0.092 | 0.098 | 0.092 |
|  | (-0.063) | (-0.063) | (-0.063) | (-0.063) | (-0.062) | (-0.063) | (-0.063) | (-0.063) |
| Occasionally | 0.126*** | 0.137*** | 0.138*** | 0.132*** | 0.128*** | 0.126*** | 0.139*** | 0.141*** |
|  | (-0.037) | (-0.037) | (-0.037) | (-0.037) | (-0.037) | (-0.037) | (-0.038) | (-0.038) |
| Often | 0.218*** | 0.241*** | 0.239*** | 0.234*** | 0.234*** | 0.221*** | 0.242*** | 0.253*** |
|  | (-0.046) | (-0.046) | (-0.046) | (-0.046) | (-0.045) | (-0.046) | (-0.046) | (-0.046) |
| Always | 0.356*** | 0.348*** | 0.364*** | 0.332*** | 0.315*** | 0.322*** | 0.359*** | 0.360*** |
|  | (-0.074) | (-0.074) | (-0.073) | (-0.074) | (-0.073) | (-0.074) | (-0.074) | (-0.074) |
| Experiencing medical disputes(ref:Never) | | | | | | | | |
| Once | 0.127** | 0.130** | 0.125** | 0.123** | 0.122** | 0.120** | 0.139*** | 0.137*** |
|  | (-0.051) | (-0.052) | (-0.052) | (-0.052) | (-0.051) | (-0.052) | (-0.052) | (-0.052) |
| Occasionally | 0.163*** | 0.183*** | 0.173*** | 0.166*** | 0.155*** | 0.163*** | 0.200*** | 0.200*** |
|  | (-0.04) | (-0.04) | (-0.04) | (-0.04) | (-0.04) | (-0.04) | (-0.04) | (-0.04) |
| Often | 0.314*** | 0.338*** | 0.288*** | 0.286*** | 0.276*** | 0.313*** | 0.352*** | 0.354*** |
|  | (-0.064) | (-0.064) | (-0.065) | (-0.065) | (-0.064) | (-0.064) | (-0.064) | (-0.064) |
| Always | 0.281 | 0.235 | 0.234 | 0.292 | 0.229 | 0.251 | 0.295 | 0.282 |
|  | (-0.203) | (-0.203) | (-0.203) | (-0.203) | (-0.201) | (-0.203) | (-0.204) | (-0.205) |
| Cons | -0.049 | -0.015 | -0.011 | -0.019 | -0.018 | -0.013 | -0.021 | -0.016 |
|  | (-0.079) | (-0.079) | (-0.079) | (-0.079) | (-0.078) | (-0.079) | (-0.08) | (-0.08) |

Note:****P* < 0.001, ***P* < 0.01, **P* < 0.05; Standard errors in parentheses.
